# 

**Published:** 1878-09

**Authors:** 


CONTENTS.

ORIGINAL.

ART. I.—Cancerous Disease of the Lungs. By J. Fowler, M. D., ........ 41
ART. II.—Abstract Proceedings Buffalo Medical Association, ........ 45
ART. III.—Surgical Cases. By C. A. Wall, B. S. 56

MISCELLANEOUS.

Observations on deep Injection of Morphia, .... 59
Antiseptic Dressing of Prof. Lister, ........ 61
Effects of Posture on Peripheral Circulation, ... 63
Diarrhœa of Children and its Treatment, ....... 65
New, Cheap and Self-generating Disinfectant.. 66
Smartweed, ........ 66
Yellow Fever Plague, ........ 67
Wanga Plant and Voudooism, ........ 67
Lactopeptine, ........ 72
Open Air Treatment, ........ 73
Metric System, ........ 73

EDITORIAL.

Are Physicians to be driven back to their old plan of furnishing their own medicines, .. 73
Items, ........ 74

BOOKS REVIEWED:
Transactions of the American Gynecological Society, ........ 77
Sources of Muscular Power. By Austin Flint, Jr. M. D., ........ 78
A Manual of Operative Surgery. By Lewis A. Stimson, B. A., M. D., ........ 79

Books and Pamphlets Received, ........ 80

